# Assessing the Risk of Invasion by Tephritid Fruit Flies: Intraspecific Divergence Matters

**DOI:** 10.1371/journal.pone.0135209

**Published:** 2015-08-14

**Authors:** Martin Godefroid, Astrid Cruaud, Jean-Pierre Rossi, Jean-Yves Rasplus

**Affiliations:** Centre de Biologie pour la Gestion des Populations, Institut National de la Recherche Agronomique, Montferriez-sur-Lez, France; University of Thessaly, GREECE

## Abstract

Widely distributed species often show strong phylogeographic structure, with lineages potentially adapted to different biotic and abiotic conditions. The success of an invasion process may thus depend on the intraspecific identity of the introduced propagules. However, pest risk analyses are usually performed without accounting for intraspecific diversity. In this study, we developed bioclimatic models using MaxEnt and boosted regression trees approaches, to predict the potential distribution in Europe of six economically important Tephritid pests (*Ceratitis fasciventris* (Bezzi), *Bactrocera oleae* (Rossi), *Anastrepha obliqua* (Macquart), *Anastrepha fraterculus* (Wiedemann), *Rhagoletis pomonella* (Walsh) and *Bactrocera cucurbitae* (Coquillet)). We considered intraspecific diversity in our risk analyses by independently modeling the distributions of conspecific lineages. The six species displayed different potential distributions in Europe. A strong signal of intraspecific climate envelope divergence was observed in most species. In some cases, conspecific lineages differed strongly in potential distributions suggesting that taxonomic resolution should be accounted for in pest risk analyses. No models (lineage- and species-based approaches) predicted high climatic suitability in the entire invaded range of *B*. *oleae*—the only species whose intraspecific identity of invading populations has been elucidated—in California. Host availability appears to play the most important role in shaping the geographic range of this specialist pest. However, climatic suitability values predicted by species-based models are correlated with population densities of *B*. *oleae* globally reported in California. Our study highlights how classical taxonomic boundaries may lead to under- or overestimation of the potential pest distributions and encourages accounting for intraspecific diversity when assessing the risk of biological invasion.

## Introduction

Controlling invasive species has become a task of utmost importance as the rate of biological invasions increases, leading to a global economic cost estimated at US$ 1.4 trillion per year, representing nearly 5% of the worldwide economy [1]. Because commercial exchanges are continuously increasing and since human activities lead to important environmental changes, numerous pest species are—and will be—susceptible to geographical expansion, thereby causing new problems for agriculture [2]. An important element of control lies in the development of powerful and accurate predictive tools, allowing a proper risk assessment [3], which, in turn, will be helpful to prevent the introduction of alien species and design cost-effective management strategies. Pest risk analyses (PRAs) address organisms' biology and ecology to discriminate potentially innocuous exotic species from harmful ones. Besides integrating the multiplicity of ecological and anthropological factors susceptible to enhance invasion risk, estimating climatic tolerances of exotic species constitutes a crucial point in PRAs. Pest risk analyses often rely on species distribution models (SDMs) when estimating the potential distribution of target species outside their current geographical range [4]. The existence of different intraspecific lineages potentially adapted to different biotic (e.g. host-plant [5]) and abiotic conditions (e.g. climatic tolerances [6, 7]) is generally ignored. However, there is increasing evidence that the origin of introduced population(s) might determine the success of an invasion [7–10]. Thus, as suggested by Peterson & Holt [11], in cases where models have been developed without taking into account the phylogeographic structure of the potential invader, invasion risk could be under- or overestimated, leading to inappropriate phytosanitary measures.

The ‘true’ fruit flies (Diptera, Tephritidae) are important agricultural pests worldwide [12]. The larvae of most species are phytophagous and attack tissues of a wide spectrum of plants, especially fruits and flowers, significantly reducing crop yields. As a result, many species are of high economic importance (especially in the genera *Bactrocera*, *Anastrepha*, *Rhagoletis* and *Ceratitis)*. In addition, several species (e.g. *Bactrocera dorsalis* (Hendel, 1912), *Bactrocera cucurbitae* (Coquillet, 1899), *Bactrocera oleae* (Rossi, 1790), *Bactrocera depressa* (Shiraki, 1933), *Ceratitis capitata* (Wiedemann, 1824), and *Ceratitis rosa* Karsch, 1887) have been successful invasive species over the past decades [13–19]. For these reasons, tephritid flies are indiscriminately listed as quarantine organisms for Europe. Several tephritid species show strong genetic structure and some are considered species complexes (e.g. the *Anastrepha fraterculus* group, the *Bactrocera dorsalis* group and the *Ceratitis fasciventris*, *C*. *rosa*, *C*. *anonae* complexes) [20–24]. Lineages can display different life history traits related to adaptations to local climate [25–29] or different host preferences [30], which make control strategies more difficult and lead to the validity of species-based PRAs to be questioned. Tephritid flies are thus good candidates to test whether the phylogeographic structure of a potential invasive pest should be considered when modeling species distribution in the context of invasion risk assessment.

In the present paper, we first used the classical species-based approach to assess the potential distribution in Europe of these economically important tephritid species. In a second step, we developed a set of models accounting the phylogeographic structure of the species.

## Material & Methods

### Species data and phylogeographic patterns

We studied six, widely distributed and economically important tephritid species, namely *Bactrocera oleae*, *Bactrocera cucurbitae*, *Anastrepha obliqua* (Macquart, 1835), *Anastrepha fraterculus* (Wiedemann, 1830), *Rhagoletis pomonella* (Walsh, 1867) and *Ceratitis fasciventris* (Bezzi, 1920). We chose these species because their phylogeographic structure has previously been described in the literature [21, 23, 31–37]. Most occurrences were collected from the scientific literature (more than 95 publications; S1 File) and online databases (e.g. Global Biodiversity Information Facility (GBIF), National Agricultural Pest Information System (NAPIS) and the BioSystematics Database of World Diptera). Doubtful or imprecise records were removed from our datasets. We assigned each occurrence to a genetic lineage according to the phylogeographic pattern described in the relevant publication (Table 1 and S2 File). Several records were assigned to more than one lineage, since several lineages occurred in the same geographical area. Some records were used in species-based models only when assignation to one lineage was challenging according to phylogeographic studies (S2 File).

**Table 1:** Tephritid fruit flies selected to assess invasion risk and integrate intraspecific diversity in species distribution models.

Species	Lineages	Symbol	Geographic distribution
*Bactrocera oleae*	All lineages	Bo_all	Africa, Asia, Europe, Americas
	Middle East lineage	Bo_me	Israel, Turkey, Cyprus, Syria
	Western Europe lineage	Bo_we	Spain, Italy, Greece, France, Morocco, Tunisia, Turkey, Algeria
	Africa lineage	Bo_af	Africa
	Asia lineage	bo_as	Pakistan
*Ceratitis fasciventris*	All lineages	Cf_all	Africa
	Western Africa lineage	Cf_we	Western Africa & Eastern Tanzania
	Eastern Africa lineage	Cf_ke	Eastern & Central Africa
*Anastrepha obliqua*	All lineages	Ao_all	Mexico, South and Central America, Caribbean
	Northern lineage	Ao_01	Central America, Caribbean, Northern Andean
	Western Mexico lineage	Ao_wm	Western & Southeastern Mexico
	South America lineage	Ao_03	South America, Northern Andean, Panama
*Anastrepha fraterculus*	All lineages	Af_all	USA, Mexico, Central America, South America
	Mexican lineage	Af_mex	Mexico & Central America
	Brazilian lineage	Af_bra	South America
	Andean lineage	Af_and	Northern Andean (high altitude)
*Rhagoletis pomonella*	All lineages	Rp_all	USA, Canada & Mexico
	USA lineage	Rp_usa	USA & Canada
	Mexican lineages	Rp_mex	Mexico
*Bactrocera cucurbitae*	All lineages	Bc_all	Asia, Africa, USA, La Réunion

The olive fruit fly *B*. *oleae* occurs in Africa, Europe and Asia and has recently invaded California and Mexico [38]. Genetic analyses suggest the Middle East region as the most likely origin of invading populations [21, 22, 38]. A deep genetic differentiation exists between Asian (var. *asiatica*), Mediterranean and African populations and closely concurs with the pattern of Quaternary differentiation of its hosts *Olea* spp. [22]. Within the Mediterranean region, microsatellite and mitochondrial markers suggested divergence between populations from the Near East (eastern Turkey, Israel, Cyprus) and populations from Western Europe and Northern Africa (Italy, Spain, Greece, France, Morocco, Algeria) [21, 22, 38, 39]. Recently, *B*. *oleae* was also detected on olive in China, but whether these populations are native or recent invaders is still a matter of debate [40]; for that reason, we did not consider the Chinese populations in our study. Given the phylogeographic pattern of *B*. *oleae*, we considered four native lineages as relevant in our study (Africa, Western Europe, Middle East and Pakistan) and one invasive population (America) (Fig A in S2 File). As the reasons for the existence of shared haplotypes among phylogeographic regions is still debated (e.g. cryptic invasions due to global olive trade or incomplete lineage sorting [21]), we deliberately ignored potential geographic overlapping among conspecific lineages.

The West Indian fruit fly *Anastrepha obliqua* occurs in the Neotropics from northern Mexico to southern Brazil as well as in the Caribbean islands. This polyphagous pest species mainly feeds on *Mangifera indica* and *Spondias* species (Anacardiaceae) [41]. Three well-supported infraspecific lineages were identified using mitochondrial markers [23]. One lineage encompasses specimens sampled across Central America, Mexico, Caribbean islands and northern Andean highlands and all records from this area were assigned to this lineage (Fig C in S2 File and Table 1). A second lineage encompasses specimens sampled mostly across Mexico west to the Sierra Madre Occidental (SMOC). However, one haplotype associated with this lineage was recovered in Southeastern Mexico (locality of Agua Blanca in Tabasco province). The occurrence of this lineage in this region is being debated, so we did not consider this record, which could probably have resulted from a recent invasion process [23]. All occurrences of *A*. *obliqua* in Western Mexico were assigned to this lineage (Table 1 and S2 File). Finally, a third lineage encompasses haplotypes recovered from South America, including the northern Andean highlands and Panama. All documented records from these regions were assigned to this lineage (Table 1 and Fig C in S2 File).

The Afrotropical *Ceratitis fasciventris* is a polyphagous fruit fly [42, 43]. Genetic structure was detected within *C*. *fasciventris* by both microsatellite data and mitochondrial markers [24, 44]. Molecular markers suggest that one infraspecific lineage occurs in western Africa and in coastal Tanzania, while a second lineage occurs in the highlands of eastern Africa (Kenya, Uganda, Ethiopia, Zambia) [24, 44]. Additionally, two clades composed of specimens from Benin and Mali/Côte d’Ivoire were highlighted by the analysis of mitochondrial sequences of specimens from Western Africa [44]. As the preliminary results showed that climatic envelopes of these two clades were broadly similar, we considered these lineages as a single set. We removed from our dataset the records of *C*. *fasciventris* in Democratic Republic of Congo, Rwanda, Namibia, Angola and Congo, since these geographic zones were not sampled in the phylogeographic studies [24, 44]. Finally, we assigned all records from Western Africa and the lowlands of Tanzania to one lineage (Table 1 and Fig B in S2 File). The records from the highlands of Eastern Africa were assigned to a second lineage (Table 1 and Fig B in S2 File).

*Anastrepha fraterculus* is considered a species complex (*AF* complex) ranging from South America to the USA [45]. Cryptic species of *A*. *fraterculus* occur in different geographic areas, displaying different host preferences and showing signals of reproductive isolation [30, 46]. Although the taxonomic status of several entities within the complex is still debated, some of them are differentiated based on genetic and morphological evidence [31, 37, 47–49]. In this study, we considered independently a "Mexican' lineage occurring across Central America from Mexico to Panama [37], an 'Andean' lineage [31, 37] occurring in the highlands of the Andean region (approximately in regions located at altitudes ranging from 1200 to 2500 meters (see [37]) and a 'Brazilian' lineage occurring in Brazil and Argentina [37, 47, 49] (Table 1 and Fig D in S2 File). We did not consider types occurring in the lowlands of Peru, Venezuela and Colombia because we lacked occurrences in these regions. As occurrences were also lacking to model the distribution of the Andean lineage, we artificially generated 1,000 additional presences at altitudes ranging between 1,200 and 2,500m to perform our SDMs. We estimated this amount of occurrences to be large enough to accurately model the distribution of species [50, 51].

The apple maggot fly, *R*. *pomonella*, is widely distributed in the USA, Canada and mountainous ranges of Mexico. This species has deep economic implications since *R*. *pomonella* recently shifted to the introduced cultivated apple (*Malus pumila* Mill.). This species encompasses at least four genetically distinct populations [32, 33, 35] displaying mating incompatibilities [52]. These populations occur respectively in the USA, the Eje Volcanico Trans Mexicano (EVTM), the Sierra Madre Oriental (SMO) and the elevated regions of Chiapas in Mexico. Because we lacked occurrences of *R*. *pomonella* in Mexico, and the preliminary results showed high climatic envelope similarity among Mexican populations, we considered all Mexican records as belonging to a 'Mexican' lineage (Table 1 and Fig E in S2 File).

When assessing the invasion risk of *B*. *cucurbitae*, we only considered the species level (Fig F in S2 File). Although genetic structure exists in *B*. *cucurbitae* [34], differentiation among geographically distant populations appears mostly as a result of human-mediated range expansions rather than ancient geographic and climatic events [53].

### Species distribution modeling

We used climatic data available from the WorldClim database version 1.4 [54] to investigate niche divergence between lineages and to model their distributions (Table 2). These climatic data are derived from temperature and rainfall annual trends between 1950 and 2000. The choice of environmental data set is crucial when modeling potential distribution in a new area [55, 56]. We consequently decided to model the distribution of fruit flies species with a strongly restricted climatic data set of moderately correlated (Pearson correlation index r < 0.7) and biologically meaningful variables that were susceptible to inducing physiological stress for these organisms. We did not include climatic descriptors depicting the annual range of temperatures and the seasonal structure of the climate because these variables probably influence the phenology of the insects rather than inducing physiological stress. We thus selected the following climatic variables: the mean temperature of the warmest month (bio5), the mean temperature of the coldest month (bio6), the precipitation of the wettest quarter (bio16) and the precipitation of the driest quarter (bio17).

**Table 2:** Bioclimatic variables used to investigate the climatic niche of tephritid fruit flies species and lineages.

Bioclimatic variables	symbol
Annual Mean Temperature	BIO1
Mean Diurnal Range	BIO2
Isothermality	BIO3
Temperature Seasonality	BIO4
Maximum Temperature of Warmest Month	BIO5
Minimum Temperature of Coldest Month	BIO6
Temperature Annual Range	BIO7
Mean Temperature of Wettest Quarter	BIO8
Mean Temperature of Driest Quarter	BIO9
Mean Temperature of Warmest Quarter	BIO10
Mean Temperature of Coldest Quarter	BIO11
Annual Precipitation	BIO12
Precipitation of Wettest Month	BIO13
Precipitation of Driest Month	BIO14
Precipitation Seasonality	BIO15
Precipitation of Wettest Quarter	BIO16
Precipitation of Driest Quarter	BIO17
Precipitation of Warmest Quarter	BIO18
Precipitation of Coldest Quarter	BIO19

We modeled the distribution of species and intraspecific entities using MaxEnt version 3.3.3 with default settings [57] available in the 'dismo' R package [58] and the boosted regression trees (BRT) [59, 60] available in the ‘gbm’ R package [61]. These two techniques were selected because they are among the best-performing approaches to model species distributions [50, 62]. The MaxEnt approach is a ‘presence-only’ method that performs well, even with a small occurrence dataset [50, 51]. This algorithm contrasts the climatic envelope experienced by the species with a set of localities randomly chosen within the background environment, where the presence of the species is unknown. The choice of background area is crucial when modeling distribution with MaxEnt and depends on the purpose of the study [63–65]. The BRT modeling technique is a machine-learning approach relying on presences and absences that can fit complex non-linear relationships and handle correlation effects between predictors [60]. All information regarding the positioning of absences, pseudo-absences and background data used to model the distribution of each species and lineage are available in Supplementary information (S1 Text).

We modeled the distribution of all Species (‘S-models’) and IntraSpecific lineages (‘IS-models’). Each model was fitted using a random subset of 80% of the relevant occurrences (training dataset) and tested with the remaining records. The predictive power of each model was assessed by measuring the area under the receiver operating characteristics (ROC) curve (AUC) [66]. The AUC is widely used in species distribution modeling because it is a threshold-independent evaluation approach that gets around the problem of subjectivity of threshold selection when assessing the accuracy of bioclimatic models. Values of AUC ranging from 0.5 (random prediction) to 1 (perfect prediction) indicate model performance better than random. The AUC of each model for *B*. *oleae* in the invaded range was also assessed with observed presences in Western America and 10000 pseudo-absences generated in Western America (i.e. in a square with longitude ranging from 122.3 to -115.4 and latitude ranging from 31.86 to 39.51).

All models were projected onto both world and Europe maps to assess the risk of biological invasion at worldwide and European scales. Since projections onto new areas may be particularly uncertain, we computed multivariate environmental similarity surface maps (MESS) [65] that describe the similarity between the climatic spaces of the training and the projected areas. Negative values of MESS indicate that the model is projected onto the area outside the climatic space where the model was fitted (model extrapolation), whilst positive values of MESS indicate model interpolation. We constructed MESS maps using occurrence and background data.

### Niche comparison

We carried out direct climate comparisons by means of Principal Component Analysis (PCA). This multivariate technique allows climate typologies to be drawn and groups of occurrences associated to similar climate descriptors to be identified [67, 68]. The differences between lineages of a species were assessed using a between-class inertia test [69] based on 999 permutations [70]. The higher the between-class inertia, the greater the difference between classes in the multivariate-space under study. The analyses were performed using the 19 bioclimatic variables from the WorldClim database (Table 2).

We investigated the degree of niche similarity between conspecific lineages by calculating Schoener’ D index [71]. This metric measures the overlap of the SDM projections, which varies from 0 for non-overlapping model predictions to 1 for complete overlap. We deliberately did not address intraspecific niche divergence using recently developed statistical approaches [72–76] because of the difficulty in delimiting different and non-overlapping background regions (i.e. pseudoabsences) in SDMs among conspecific lineages. The uncertainty related to the positioning of pseudoabsences may, indeed, have large effects on SDM predictions and consequently alter the reliability of tests of climatic niche divergence [72, 77].

## Results

### Realized climatic niche similarity between conspecific lineages

**Fig 1:**
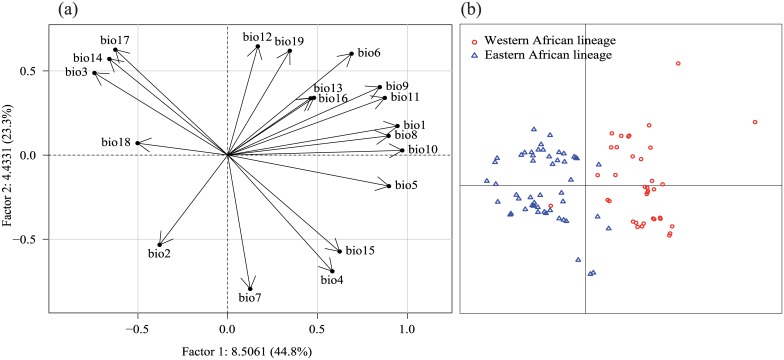
Principal component analysis (PCA) performed on 19 bioclimatic variables extracted from the Worldclim database [54] for *Ceratitis fasciventris* occurrences (see Table 2 for symbol meanings). These multivariate analyses draw the bioclimatic envelopes of the different phylogeographic lineages belonging to *C*. *fasciventris*. Circles of correlation (a) and factorial scores of records (b) are shown. Percentages of variance explained by each PCA axis are indicated in correlation circle.

Between-class PCA analyses showed significant climatic envelope divergence for all of the species considered (values of inertia are available in S1 Table). High divergence was observed among conspecific lineages of *C*. *fasciventris* (p<0.001) and *A*. *fraterculus* (p<0.001) according to temperature variables such as the mean annual temperature (bio1), the mean temperature of the warmest months (bio5, bio10), and the mean temperature of the coldest months (bio6, bio11) (Fig 1 and Fig B in S3 File). The 'Andean' lineage of *A*. *fraterculus* occurred in regions characterized by high precipitations (bio14, bio17) and fresh maximal temperatures (bio5, bio10).

Conspecific lineages of *R*. *pomonella* mainly differed (p<0.001) according to minimal temperatures (bio6, bio11), temperature annual range (bio2, bio3, bio4, bio7) and maximal precipitations (bio13, bio16) (Fig C in S3 File). However, conspecific lineages of *R*. *pomonella* occur in areas with similar maximal temperatures (bio5, bio10).

No strong differences were observed between the Ao_01 and Ao_03 lineages of *A*. *obliqua* (p<0.001) along the two first PCA axes performed on the climatic data corresponding to occurrence points (Fig A in S3 File). However, the climatic envelope of population Ao_02 was different from that of populations Ao_01 and Ao_03 according to precipitations (bio12, bio13, bio15) and maximal temperatures (bio5, bio10).

**Fig 2:**
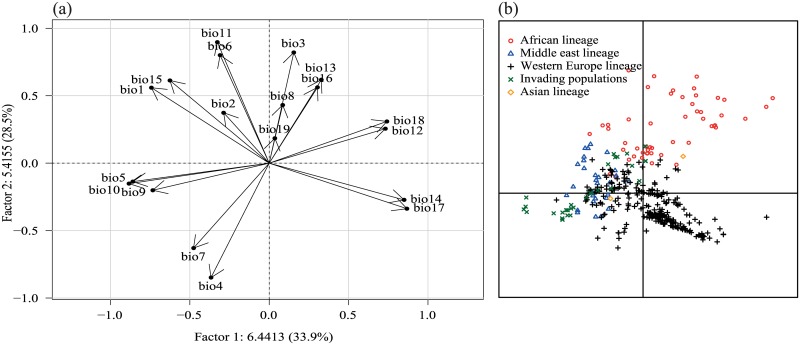
Principal component analysis (PCA) performed on 19 bioclimatic variables extracted from the Worldclim database [54] for *Bactrocera oleae* occurrences (see Table 2 for symbol meanings). These multivariate analyses draw the bioclimatic envelopes of the different phylogeographic lineages belonging to *B*. *oleae* as well as the invading populations in Americas. Circles of correlation (a) and factorial scores of records (b) are shown. Percentages of variance explained by each PCA axis are indicated in correlation circle.

Lineages of *B*. *oleae* (p<0.001) were opposed on the first PCA axis by warmest temperatures (bio5, bio10; Fig 2) and by precipitation descriptors such as the mean annual temperature (bio12), the precipitation of the driest month (bio14) and the precipitations of the warmest quarter (bio 18). On the second PCA axis, populations were opposed by the temperature annual range (bio7, bio4) and by the minimum temperature (bio6, bio11; Fig 2). The bioclimatic envelope of *B*. *oleae* in the invaded range was different from the other lineages with respect to maximal temperatures (bio5, bio10) and minimal temperatures (bio6, bio11).

### Potential distributions of tephritid species

**Fig 3:**
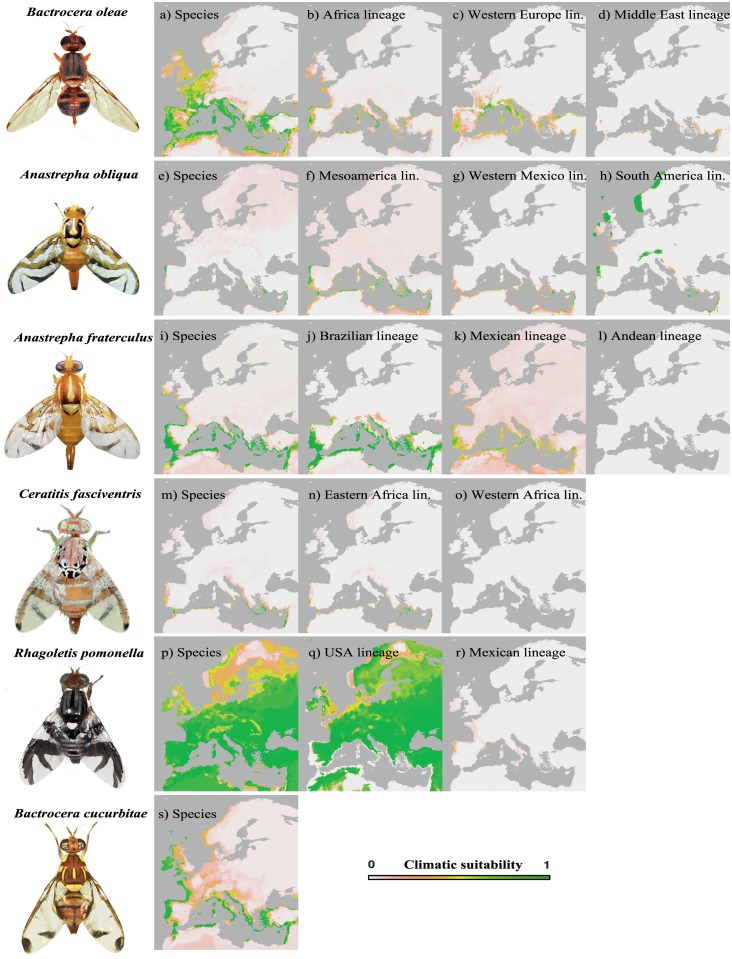
Projections of climatic suitability for six tephritid species and intraspecific lineages in Europe ((a-d) *Bactrocera oleae*, (e-h) *Anastrepha obliqua*, (i-l) *Anastrepha fraterculus*, (m-o) *Ceratitis fasciventris*, (p-r) *Rhagoletis pomonella* and (s) *Bactrocera cucurbitae*) as predicted by species distribution models (SDMs). Species- and lineage-based SDMs were performed using the Boosted Regression Trees (BRT) [60]. Climatic suitability is shown by a color gradient, which goes from green (high probability) to light orange (low probability).

Our SDM models displayed relatively high values of cross-validated AUC ranging from 0.76 to 0.98 (S2 Table). The BRT models globally displayed higher values of cross-validated AUC than MaxEnt models. The distribution of suitable climatic conditions for *C*. *fasciventris*, *A*. *fraterculus*, *B*. *cucurbitae* and *A*. *obliqua* predicted by SDM models encompassed most of the tropical and subtropical areas of the world and a few regions with Mediterranean or temperate climate (S4 and S5 Files). However, the distribution of suitable conditions for *B*. *oleae* and *R*. *pomonella* mainly encompassed temperate regions of the world (Figs A and E in S4 and S5 Files). The potential distributions of all tephritid species encompassed areas in Europe (Fig 3 and S5 File). Both *R*. *pomonella* and *B*. *oleae* displayed the larger extents of suitable climatic conditions in Europe (Fig 3 and Figs A and E in S5 File). Both *C*. *fasciventris* and *A*. *obliqua* showed a small potential distribution range in Europe (Fig 3 and Figs B and C in S5 File). Maxent maps of *C*. *fasciventris* predicted large potential areas in Europe (e.g. in the United Kingdom and Scandinavia; Fig B in S5 File). However, MESS maps indicated that large model extrapolation occurs when predicting the potential distribution of *C*. *fasciventris* in Northern Europe. We argue that the BRT prediction is the most reliable for *C*. *fasciventris* since the MaxEnt model predicts climatic suitability in implausible areas in northern Europe (such inconsistency appears common with the MaxEnt algorithm, see [78]). Both *A*. *fraterculus* and *B*. *cucurbitae* displayed a larger extent of suitable climatic conditions with respect to *A*. *obliqua* and *C*. *fasciventris* (Fig 3 and Figs D and F in S5 File).

We observed strong differences in the modeled distributions among conspecific lineages of *C*. *fasciventris*, *A*. *fraterculus* and *R*. *pomonella* (Fig 3, S4 and S5 Files; Table 3). However, conspecific lineages of *B*. *oleae* and *A*. *obliqua* showed similar but not identical modeled distributions (Fig 3 and Figs A and C in S4 and S5 Files; Table 3).

**Table 3:** Measures of species distribution models overlapping among phylogeographic lineages of tephritid fruit flies. We calculated projection overlapping by calculating the Schoener' D index, which range from 0 (no overlapping) to 1 (perfect overlapping).

Species	Lineage 1	Lineage 2	Schoener' Index	
			BRT	MaxEnt
*Ceratitis fasciventris*	lineage Eastern Africa	lineage Western Africa	0.46	0.23
*Rhagoletis pomonella*	lineage USA	lineage Mexico	0.1	0.13
*Anastrepha fraterculus*	lineage Mexico	lineage Brazil	0.8	0.64
	lineage Mexico	lineage Andean	0.13	0.12
	lineage Brazil	lineage Andean	0.09	0.11
*Anastrepha obliqua*	lineage Central America	lineage Western Mexico	0.91	0.55
	lineage Central America	lineage South America	0.86	0.59
	lineage South America	lineage Western Mexico	0.87	0.29
*Bactrocera oleae*	lineage Africa	lineage Western Europe	0.32	0.55
	lineage Africa	lineage Middle East	0.25	0.42
	lineage Western Europe	lineage Middle East	0.29	0.48

**Fig 4:**
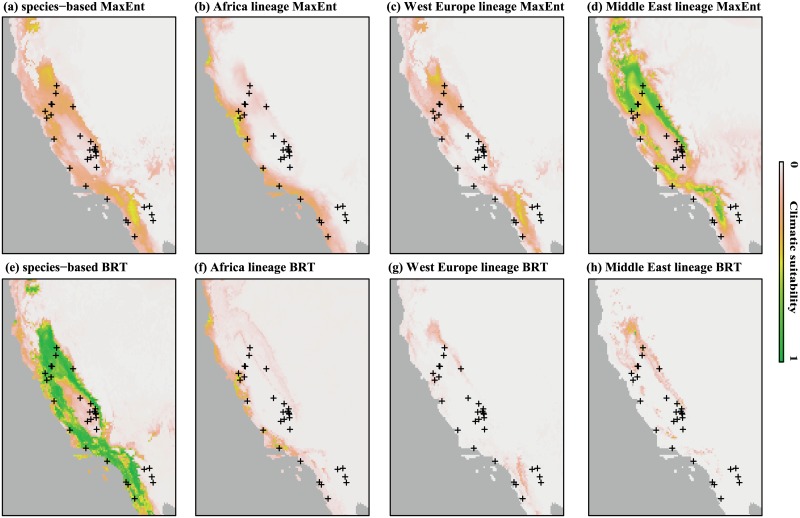
Projections of species- and lineage-based models (MaxEnt and Boosted Regression Trees (BRT) for *Bactrocera oleae* in the invaded range in Americas. Black crosses represent the occurrence of the olive fruit fly. Models were calibrated independently for the species and the different conspecific lineages. Climatic suitability is shown by a color gradient, which goes from green (high probability) to light orange (low probability).

We suggest that the MaxEnt algorithm provides the most reliable potential distribution of the Ao_03 lineage since the BRT model predicts climatic suitability in implausible areas (cold mountainous regions of Europe and Greenland). The distributions of suitable climatic conditions predicted by the BRT and MaxEnt S-models for *B*. *oleae* were broadly congruent. They matched the geographic distribution of mild temperate climates and Mediterranean-climate regions where native and cultivated olives occur (e.g. California, Australia, Southeast China, Argentina, Chile, Mexico, Mediterranean area), while also encompassing colder temperate regions. Our SDMs also predicted climatic suitability for *B*. *oleae* in temperate regions (the United Kingdom, Northern France, Belgium, south-eastern United states), where olive trees are not currently cultivated. The records of the genetically distinct lineage occurring in Pakistan were well predicted by S-models. Conspecific lineages of *B*. *oleae* showed slightly different modeled distributions in the invaded range. The models predicted higher climatic suitability in coastal region of California for the African lineage while inland regions were predicted as more suitable for Western European and Middle Eastern lineages (Fig 4). The AUC values displayed by MaxEnt and BRT S-models for *B*. *oleae* in the invaded range were 0.65 and 0.68 respectively indicating model performance better than random. The AUC values in the invaded range displayed by the species-based BRT model was higher than those displayed by lineages-based BRT models (0.55, 0.46 and 0.57 for African, Western European and Middle Eastern lineages respectively). The AUC value in the invaded range displayed by species-based MaxEnt model was higher than those displayed by western European and Middle Eastern lineages-based BRT models (0.51 and 0.60 respectively) but slightly inferior to AUC displayed by African lineage-based MaxEnt model (0.73).

## Discussion

### Tephritid fruit flies: a current major threat with potential for future expansion

Tephritid fruit flies are among the most destructive fruit pests and constitute a major threat to worldwide agriculture. Because many species have invaded new regions where they cause severe damage to fruit and other crops [79], the group is under surveillance by biosecurity agencies to design cost-effective management strategies. In addition, investigating the climatic suitability of Europe for mainly tropical tephritid should help to uncover why paradoxically few species have invaded areas in Europe and in Northern Africa compared to other geographic regions such as California [19]. Our results show that all target tephritid species under investigation could potentially find suitable climate conditions in Europe, albeit to different degrees (Fig 3 and S5 File). Three groups of species may be distinguished according to their potential distribution range in Europe:

1. *C*. *fasciventris* and *A*. *obliqua*, which are restricted to low and mid-elevated areas of tropical regions and consequently display a low probability of being established in European countries (Fig 3 and Figs B and C in S5 File). Only areas with mild winters (e.g. the Atlantic coasts of Portugal and Spain, southern Greece, southern Turkey and Near East region) appeared to be suitable for these species (Fig 3). Cold stress would likely be the most limiting climatic variable for the long-term establishment of these flies in Europe. Indeed, *A*. *obliqua* has been reported to be less abundant at high altitudes compared to its close relatives (e.g. *A*. *fraterculus*, *A*. *ludens*) and *Ceratitis* species (e.g. *C*. *capitata*) [80, 81], while *C*. *fasciventris* is absent from colder regions in South Africa where a congeneric species *C*. *rosa* occurs [82]. However, we cannot exclude that invasive fruit flies might overwinter as larvae within infested host fruit as reported for *C*. *capitata* [83], and consequently could become established in colder areas.
2. *Anastrepha fraterculus* and *B*. *cucurbitae* displayed a larger potential distribution in Europe (Fig 3 and Figs D and F in S5 File). In their native range, these species occur in tropical lowlands and in colder elevated regions, suggesting that low winter temperatures may be less constraining than for *C*. *fasciventris* and *A*. *obliqua*. For example, *A*. *fraterculus* is sympatric with *C*. *capitata* in South America and is thus expected to display climatic tolerances allowing it to become established in parts of temperate Europe, which is also the case for *C*. *capitata* [84, 85]. In the case of *A*. *fraterculus*, we deliberately calibrated SDMs with occurrences located in dry areas where the species is associated with human settlements (e.g. irrigated areas in the 'Chaco' region and in dry highlands of the Argentinean province of Jujuy). We acknowledge that these records might lead to an overestimation of the potential distribution of *A*. *fraterculus* into dry regions, since climatic layers do not reflect microclimates resulting from human activities. We nevertheless added these records to our SDMs to avoid omission error [84]. Indeed, irrigated agriculture is performed in many dry regions of the world and might allow tephritid colonization despite apparently unfavorable macroclimatic conditions. These results also highlighted that *B*. *cucurbitae* might expand its invaded range towards southern parts of Africa where it has not yet been detected (Fig F in S4 and S5 Files).
3. *Rhagoletis pomonella* and *B*. *oleae* occur only in temperate regions and in some tropical highlands. Apart from very cold regions, the potential distribution of *R*. *pomonella* covers all of Europe (Fig 3 and Figs A and E in S5 File). Since it naturally occurs in the Mediterranean basin, *B*. *oleae* does not constitute a new threat for Europe and we will not discuss its potential distribution in detail.

### Niche variation among conspecific lineages

Although there is increasing evidence that climatic niche variation among conspecific lineages may have strong implications in invasion risk management [7, 9, 10], such intraspecific variation in tephritid fruit flies has been poorly investigated so far (but see [25]). We illustrate here how intraspecific structure of widely distributed pests is, in some cases, associated with strong climate envelope divergence. Three fruit flies (*C*. *fasciventris*, *A*. *fraterculus*, *R*. *pomonella*) highly differ in realized climatic niche and consequently display different potential distributions (Fig 3). Interestingly, models predict one lineage of *C*. *fasciventris* to be more susceptible to expanding its geographic range into Europe. Similarly, parts of the Mediterranean Basin are predicted to be suitable for the Mexican and Brazilian lineages of *A*. *fraterculus* but not for the Andean lineage (Fig 3 and Fig D in S4 File). However, we detected no marked climate divergence among lineages of *A*. *obliqua* (Fig A in S3 File). The distributions of conspecific lineages of *A*. *obliqua* appear to be constrained by cold temperatures in Mexico as well as in South America, meaning that conspecific lineages display similar potential distributions (Fig 3 and Fig C in S4 File). Similarly, relatively high overlapping of SDM projections was observed among lineages of *B*. *oleae*.

Among these species, we only addressed the invasion of *B*. *oleae* since the origin of invading populations of the other target fruit flies under study is still unknown. Our species-based climatic suitability maps indicate low climatic suitability in mountainous ranges and in southern and central California, as previously reported [86]. Species-based and African lineage-based models best explain the invaded range of *B*. *oleae*, suggesting that no marked ecological divergence is associated with intraspecific genetic structure of this pest and invasive populations might have potentially originated from every location in its entire native range. In addition, no SDMs (species- and lineage-based approaches) predict the entire invasive range as climatically suitable for *B*. *oleae*, suggesting that, given the monophagy in this fly, host distribution mainly governs its distribution, as reported for other invasive specialist phytophagous insects [87]. Interestingly, our species-based models predict high climatic suitability in coastal region of California and in Sacramento valley where high *B*. *oleae* population densities are reported [15, 88]. However, SDMs predict low climatic suitability in San Joaquin Valley and in southeastern counties where lower population densities of *B*. *oleae* are reported probably because of the extremely hot summer temperatures that occur in these regions [89]. This case highlights how lowering the taxonomic resolution may lead, in some cases, to an increase of omission error in PRA and underlines why such approaches still need to be interpreted with caution by risk analysts.

#### Perspectives and pitfalls for plant biosecurity purposes

Niche divergence exists among conspecific lineages in several of these pests (e.g. host preferences in *AF* complex, see [30]). However, addressing climatic niche divergence among organisms is complex [90] and we acknowledge that additional data are required to claim differences in invasion risk among conspecific lineages with respect to climate. Other studies showed the poor performance of lineage-based models to predict the infraspecific identity of invasive populations [91–93] and suggested the existence of a cryptic niche conservatism that may explain, in some cases, the poor predictive power of lineage-based models. In other words, these studies highlighted that differences in the *realized* climatic niche [94] do not always reflect differences in physiological tolerances (the *fundamental* climatic niche [94]). Realized climatic niche divergence may reflect non-evolutionary mechanisms, since the geographic range of populations/lineages can also be shaped by various biotic and historical factors in addition to their unique physiological tolerances [94]. As most species, to our knowledge, do not show intraspecific divergence in their diet (with the exception of the lineages of *A*. *fraterculus* occurring in Mesoamerica and in Brazil and Argentina), we suggest that hosts do not artificially inflate climatic niche divergence signals among the lineages under study. In this context, several recent studies underlined the need to increase the taxonomic resolution in PRA to avoid underestimation of invasion risk and capture the risks of niche shifts during the invasion process [95, 96]. Moreover, as phylogeographic and taxonomic studies are usually based on neutral genetic markers, we lack evidence for genetic adaptations of lineages to particular climatic conditions and the role of phenotypic plasticity in shaping geographic distributions still needs to be addressed. Testing the predictive power of such SDMs usually involves *a posteriori* analyses of successful invasions [4]; however, data were unfortunately lacking to test the validity of our approaches with most of the species under scrutiny.

Such approaches open up new perspectives for the control of exotic species. There is now clear evidence that a climatic niche evolves over moderate evolutionary times, as for those involved in phylogeographic divergence and speciation [97, 98] with crucial consequences in invasion context [7, 9]. Our study illustrates that the intraspecific structure of widely distributed pests played an important role in pest risk analysis and should be considered when assessing quarantine status. Nowadays, the European list of quarantined organisms encompasses all non-European tephritids and does not consider taxonomic entities below the species level. Given the costs of invasive species management and quarantine measures, predicting with more accuracy the potential distribution of intercepted propagules might generate substantial economic benefits [99]. In addition, rapid and accurate molecular tools (e.g. barcoding) are now available for biosecurity agencies to identify intercepted propagules to species and even lineage levels [100–103]. The availability of such tools consequently holds great promise for the design of cost-effective control strategies against exotic species.

## Supporting Information

S1 File Occurrence data used in this study and list of scientific publications where fruit flies occurrences were retrieved. (XLS)

S2 File Distribution of phylogeographic lineages of six tephritid fruit flies. (A) *Bactrocera oleae*, (B) *Ceratitis fasciventris*, (C) *Anastrepha obliqua*, (D) *Anastrepha fraterculus*, (E) *Rhagoletis pomonella*, (F) *Bacrocera cucurbitae*.
(DOCX)

S3 File Principal component analysis on climatic data extracted from lineage occurrences. (A) *Anastrepha obliqua*, (B) *Anastrepha fraterculus*, (C) *Rhagoletis pomonella*.
(DOCX)

S4 File Worldwide projections of MaxEnt predictions for six tephritid fruit flies. (A) *Bactrocera oleae*, (B) *Ceratitis fasciventris*, (C) *Anastrepha obliqua*, (D) *Anastrepha fraterculus*, (E) *Rhagoletis pomonella*, (F) *Bacrocera cucurbitae*.
(DOCX)

S5 File Worldwide projections of BRT predictions for six tephritid fruit flies. (A) *Bactrocera oleae*, (B) *Ceratitis fasciventris*, (C) *Anastrepha obliqua*, (D) *Anastrepha fraterculus*, (E) *Rhagoletis pomonella*, (F) *Bacrocera cucurbitae*.
(DOCX)

S1 Table Inertia values displayed by between-class analyses. (DOCX)

S2 Table Area under the receiver operating characteristics (ROC) curve (AUC) for each model. (DOCX)

S1 Text Positioning of absences and background data in SDM of six tephritid fruit flies. (DOCX)
