# Supplementary material for: Assessing the Risk of Invasion by Tephritid Fruit Flies: Intraspecific Divergence Matters
Source: PLoS One. 2015 Aug 14;10(8):e0135209. doi: 10.1371/journal.pone.0135209 (PMC4537207; doi:10.1371/journal.pone.0135209)

**S2 File**: Occurrence records of lineages of tephritid fruit flies

(a) *Bactrocera oleae*


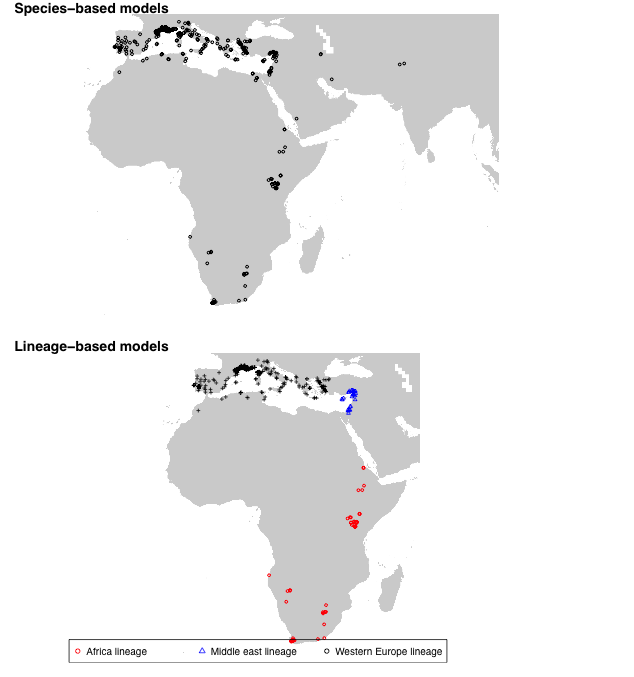


(b) *Ceratitis fasciventris*


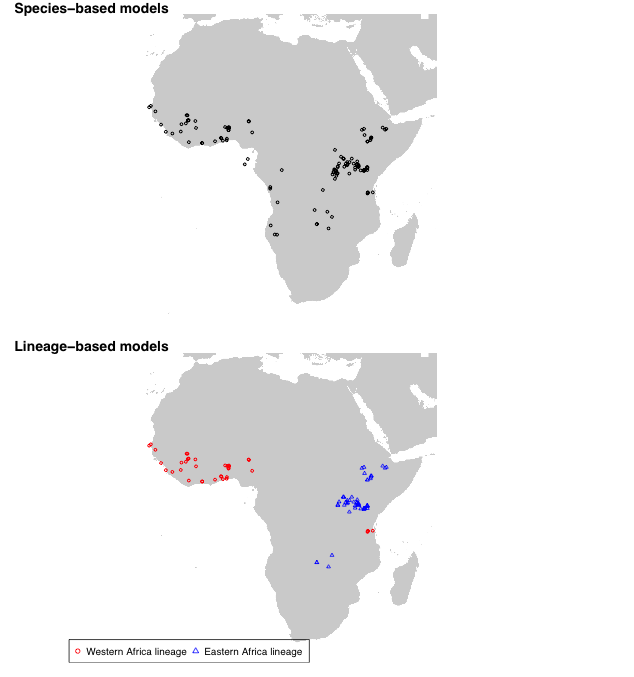


(c) *Anastrepha obliqua*


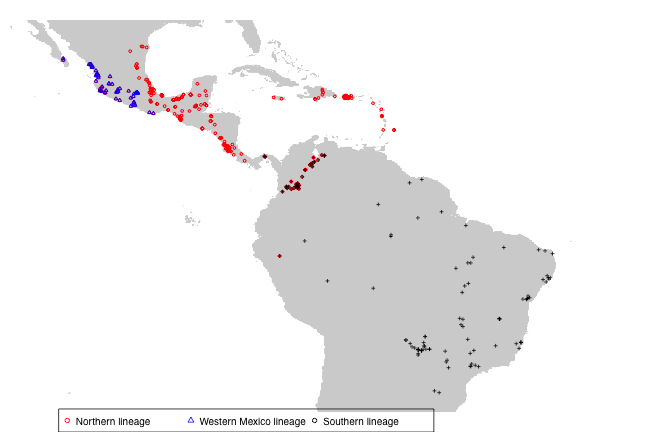


(d) *Anastrepha fraterculus*


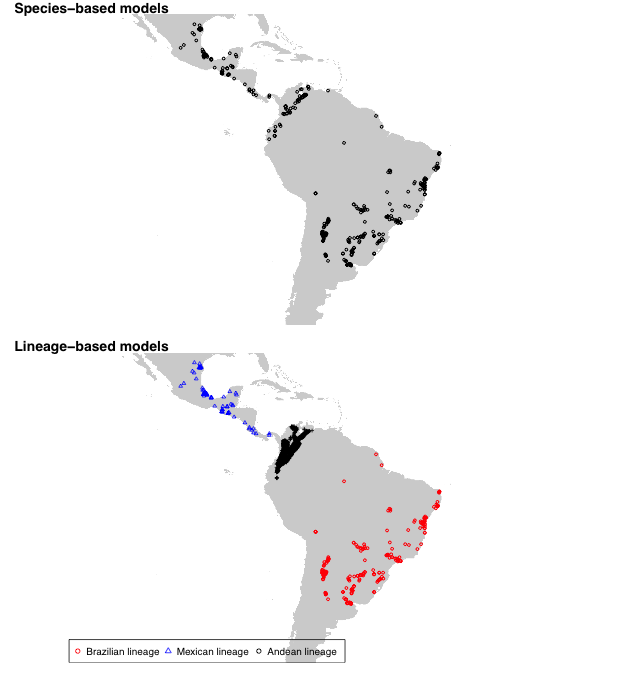


(e) *Rhagoletis pomonella*


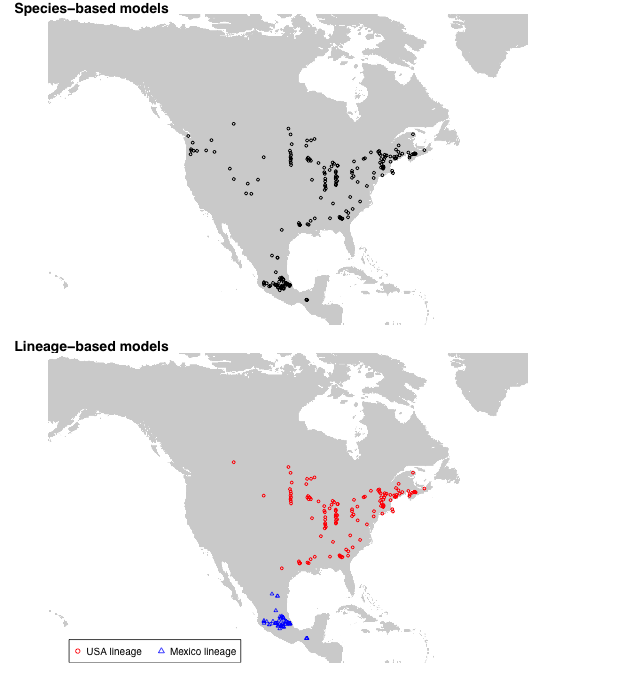


(f) *Bactrocera cucurbitae*


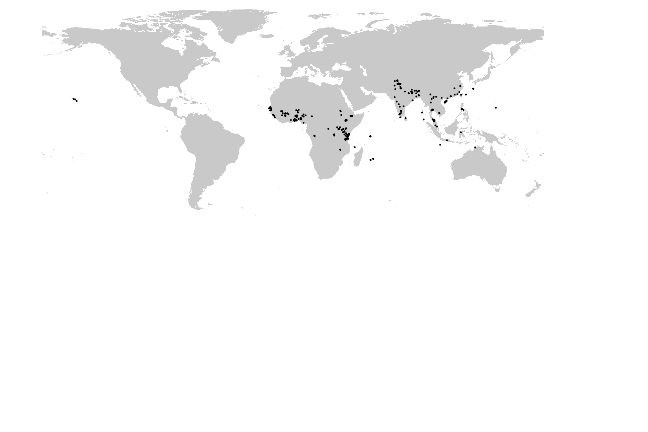

Supplement: S2 File — (A) Bactrocera oleae, (B) Ceratitis fasciventris, (C) Anastrepha obliqua, (D) Anastrepha fraterculus, (E) Rhagoletis pomonella, (F) Bacrocera cucurbitae. (DOCX) [file pone.0135209.s002.docx]
