# Supplementary material for: Assessing the Risk of Invasion by Tephritid Fruit Flies: Intraspecific Divergence Matters
Source: PLoS One. 2015 Aug 14;10(8):e0135209. doi: 10.1371/journal.pone.0135209 (PMC4537207; doi:10.1371/journal.pone.0135209)

**S3 File**: Principal component analysis on climatic data extracted from tephritid fruit flies lineages occurrences

(a) *Anastrepha obliqua*


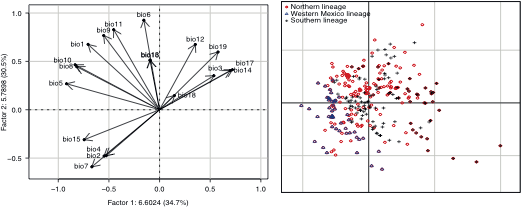


(b) *Anastrepha fraterculus*


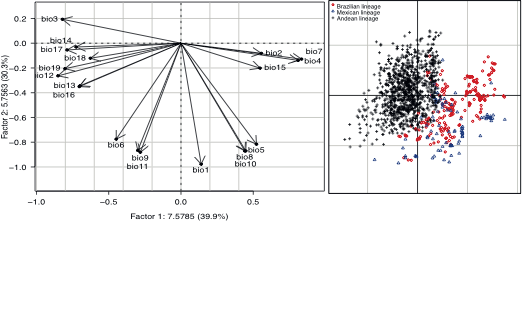


(c) *Rhagoletis pomonella*


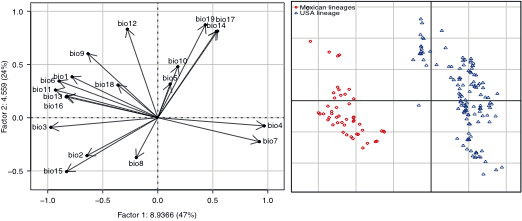

Supplement: S3 File — (A) Anastrepha obliqua, (B) Anastrepha fraterculus, (C) Rhagoletis pomonella. (DOCX) [file pone.0135209.s003.docx]
