# Supplementary material for: Assessing the Risk of Invasion by Tephritid Fruit Flies: Intraspecific Divergence Matters
Source: PLoS One. 2015 Aug 14;10(8):e0135209. doi: 10.1371/journal.pone.0135209 (PMC4537207; doi:10.1371/journal.pone.0135209)
Supplement: S1 Table — (DOCX) [file pone.0135209.s006.docx]

**Table S1:** Inertia values displayed by between-class analyses

| Species | Inertia |
| --- | --- |
| *Anastrepha fraterculus* | 0.28 |
| *Anastrepha obliqua* | 0.09 |
| *Ceratitis fasciventris* | 0.33 |
| *Bactrocera oleae* | 0.36 |
| *Rhagoletis pomonella* | 0.43 |
