# Supplementary material for: Assessing the Risk of Invasion by Tephritid Fruit Flies: Intraspecific Divergence Matters
Source: PLoS One. 2015 Aug 14;10(8):e0135209. doi: 10.1371/journal.pone.0135209 (PMC4537207; doi:10.1371/journal.pone.0135209)
Supplement: S2 Table — (DOCX) [file pone.0135209.s007.docx]

**Table S2:** Area under the receiver operating characteristics (ROC) curve (AUC) calculated for species distribution models calibrated for six tephritid fruit flies.

| Taxonomic entity | Model | |
| --- | --- | --- |
|  | BRT | MaxEnt |
| *Bactrocera oleae* all lineages | 0.97 | 0.97 |
| *Bactrocera oleae* lineage Africa | 0.90 | 0.98 |
| *Bactrocera oleae* lineage Western Europe | 0.99 | 0.98 |
| *Bactrocera oleae* lineage Middle East | 0.93 | 0.99 |
| *Ceratitis fasciventris* all lineages | 1.00 | 0.82 |
| *Ceratitis fasciventris* lineage Eastern Africa | 0.99 | 0.99 |
| *Ceratitis fasciventris* lineage Western Africa | 1.00 | 0.81 |
| *Rhagoletis pomonella* all lineages | 1.00 | 0.91 |
| *Rhagoletis pomonella* lineages USA | 1.00 | 0.92 |
| *Rhagoletis pomonella* lineages Mexico | 0.99 | 0.99 |
| *Anastrepha fraterculus* all lineages | 1.00 | 0.76 |
| *Anastrepha fraterculus* lineage Mexico | 1.00 | 0.80 |
| *Anastrepha fraterculus* lineage Brazil | 1.00 | 1.00 |
| *Anastrepha fraterculus* lineage Andean | 1.00 | 1.00 |
| *Anastrepha obliqua* all lineages | 0.98 | 0.77 |
| *Anastrepha obliqua* lineage Central America | 1.00 | 0.88 |
| *Anastrepha obliqua* lineage Western Mexico | 1.00 | 0.98 |
| *Anastrepha obliqua* lineage South America | 1.00 | 0.79 |
| *Bactrocera cucurbitae* all lineages | 1.00 | 0.91 |
