# Supplementary material for: Assessing the Risk of Invasion by Tephritid Fruit Flies: Intraspecific Divergence Matters
Source: PLoS One. 2015 Aug 14;10(8):e0135209. doi: 10.1371/journal.pone.0135209 (PMC4537207; doi:10.1371/journal.pone.0135209)
Supplement: S1 Text — (DOCX) [file pone.0135209.s008.docx]

**S1 Text:** Positioning pseudoabsences in species distribution models to predict the potential distribution of six tephritid fruit flies species and associated lineages

**(a) *Bactrocera oleae***

*Species based SDM and all lineages-based SDMs*

Maxent: 10,000 background data generated across entire Africa and Europe

BRT: 1000 absences generated across entire Africa and Europe

***(b) Ceratitis fasciventris***

- *Species based SDM:*

MaxEnt: 10,000 background data generated in Africa in regions located at a latitude below 20°N.

BRT: 150 absences generated across Africa in Sahara desert, in high altitude areas (altitude above 3000 meters) and in South Africa where *C. rosa* occurs (see De Villiers et al., 2013).

- Lineage Western Africa

MaxEnt: 10,000 background data generated in Africa in regions located at a latitude below 20°N.

BRT: We generated 150 absences across Africa in Saharan desert and in South Africa where *C. rosa* occurs (see De Villiers et al., 2013). Presence records of the eastern lineage were considered as absences when fitting this SDM.

- Lineage Eastern Africa

MaxEnt: 10,000 background data generated in Africa in regions located at a latitude below 20°N.

BRT: We generated 150 absences in Africa in Saharan desert and in South Africa where *C. rosa* occurs (see De Villiers et al., 2013). Presence records of the Western lineage were considered as absences when fitting this SDM.

(c) ***Anastrepha obliqua***

- *Species based SDM:*

MaxEnt: 10,000 background data generated in Americas from Mexico (latitude below 30°N) to Southern Argentina.

BRT: 150 absences generated in high altitude areas of Mexico (altitude above 2,300 meters) in Northern Mexico (latitude between 26°N and 30 °N) and southern regions of South America (latitude below 30°N).

- Lineage Ao_01

MaxEnt: 10,000 background data generated across Central and South America from Mexico (latitude below 30°N) to northern regions of South America (located between latitudes of 15°C S and 40°N)

BRT: 150 absences generated in high altitude areas of Mexico (altitude above 2,300 meters) in Northern parts of Mexico (latitude between 26°N and 30°N).

- Lineage Ao_wm

MaxEnt: 10,000 background data generated in Southern America from Mexico (latitude below 30°N) to Northern regions of South America (located between latitudes of 15°S and 40°N)

BRT: 150 absences generated in high altitude areas of Mexico (altitude above 2,300 meters) in Northern Mexico (latitude between 26°N and 30 °N).

- Lineage Ao_03

MaxEnt: 10,000 background data generated in South America (located between latitudes of 10°S and 50°N)

BRT: 150 absences generated in southern regions of South America (latitude below 30°N and longitude > -70°).

(d) ***Anastrepha fraterculus***

- *Species based SDM:*

MaxEnt: 10,000 background data generated in Americas from Mexico (latitude below 30°N) to Patagonia.

BRT: 150 absences generated in high altitude areas of Mexico and Central America (altitude above 2,800 meters,) in dry and high altitude regions of Eastern Argentina and Chile (latitude between 20°S and 30°S/longitude between 69.98°W and 67.72°W) and in southern regions of South America (latitude below 38°S).

- Lineage 'Mexico'

MaxEnt: 10,000 background data generated in Central America and in Mexico (latitude between 5°N and 30°N).

BRT: 150 absences generated in high altitude areas of Mexico and Central America (altitude above 2,800 meters, in dry and high altitude regions of Eastern Argentina and Chile (latitude between -20° and -30°/longitude between -69.98° and -67.72°) and in southern regions of South America (latitude below -38°).

- Lineage 'Andean'

MaxEnt: 10,000 background data generated in Central America, Mexico and Southern America (latitude below 30°N).

BRT: 10,000 background data generated in Central America, Mexico and Southern America (latitude below 30°N).

- Lineage 'Brazilian'

MaxEnt: 10,000 background data generated in South America (latitude below 5°N).

BRT: 150 absences generated in dry and high altitude regions of Eastern Argentina and Chile (latitude between -20° and -30°/longitude between -69.98° and -67.72°) and in southern regions of South America (latitude below -38°).

(e) ***Rhagoletis pomonella***

- *Species based SDM:*

MaxEnt: 10,000 background data generated in North America and Mexico (latitude above 12°N)

BRT: 150 absences generated in lowlands of Central America and Mexico (altitude below 1,000 meters) in highlands of Mexico (altitude above 3,300 meters), in cold regions of northern USA and southeastern Canada (latitude between 47°N and 47°N/ longitude between 85°W and 75°W) and in dry lowlands of eastern Mexico (state of Baja California).

- Lineage 'USA'

MaxEnt: 10,000 background data generated in North America and Mexico (latitude above 12°N)

BRT: 150 absences generated in lowlands of Central America and Mexico (altitude below 1,000 meters) in highlands of Mexico (altitude above 3,300 meters), in cold regions of northern USA and southeastern Canada (latitude between 47°N and 47°N/ longitude between 85°W and 75°W) and in dry lowlands of eastern Mexico (state of Baja California).

- Lineage 'Mexico'

MaxEnt: 10,000 background data generated in North America and Mexico (latitude above 12°N)

BRT: 10,000 background data generated in North America and Mexico (latitude above 12°N)

(f) ***Bactrocera cucurbitae***

MaXent: MaxEnt: 10,000 background data generated in Asia and Oceania (latitude between 50°S and 72°N/longitude between 122°E and 140°E)

BRT: 150 absences generated in dry central Australia (latitude between 27°S and 20°S/longitude between 70°E and 150°E), in northern parts of Asia (latitude between 28°N and 60°N/longitude between 81°E and 110°E.
